# IL-1β modulates inflammatory response of human bone marrow-derived MSCs and neutrophil recruitment in vitro via NF-kB-associated signaling

**DOI:** 10.1186/s13287-026-05029-x

**Published:** 2026-04-24

**Authors:** Nour Hassaan, Tobias Schmidt, Zackarias Söderlund, Dimitrios Kalafatis, Lisa I. Påhlman, Stefan Scheding, Daniel J. Weiss, Robin Kahn, Sara Rolandsson Enes

**Affiliations:** 1 Department of Experimental Medical Science, Faculty of Medicine, Lund University, BMC C12, 221 84 Lund, Sweden; 2 Department of Rheumatology, Institute of Clinical Sciences Lund, Lund University, Lund, Sweden; 3 WCMM Wallenberg Centre for Molecular Medicine, Lund University, Lund, Sweden; 4 Department of Pediatrics, Institute of Clinical Sciences Lund, Lund University, Lund, Sweden; 5 Division of Infectious Diseases, Skåne University Hospital Lund, Lund, Sweden; 6 Department of Clinical Sciences Lund Science, Division of Infection Medicine, Lund University, Lund, Sweden; 7 Division of Molecular Hematology and Stem Cell Centre, Lund University, Lund, Sweden; 8 Department of Hematology, Skåne University Hospital, Lund, Sweden; 9 Department of Medicine, Larner College of Medicine, University of Vermont, Burlington, VT USA

**Keywords:** ARDS, MSC, IL-1beta, RNA-seq, Cell-based therapy, NF-kB signaling, Neutrophil

## Abstract

**Background:**

The variable clinical outcomes of mesenchymal stromal cell (MSC)-based therapy in acute respiratory distress syndrome (ARDS) are attributed to a variety of factors, including host microenvironmental factors. Interleukin-1β (IL-1β) has been linked to the development and progression of ARDS, and we have previously found that IL-1β could be used to predict MSC activation in vitro. However, the exact mechanisms through which IL-1β alters the MSC function and its interaction with the host immune cells remains unknown. Therefore, the aim of this study was to assess how IL-1β alters MSC function, with a specific focus on MSC-neutrophil interaction.

**Methods:**

Human bone marrow-derived MSCs were exposed to 20 ng/ml IL-1β for 1 or 24 h. Following exposure, MSCs were analyzed using bulk RNA sequencing and key secretome proteins were measured in their conditioned medium. A transwell culture system was used to evaluate the neutrophil recruitment capacity of IL-1β-exposed MSCs, with or without NF-kB inhibition. MSCs exposed to serum free medium were used as controls in all experiments.

**Results:**

The sequencing data revealed that genes involved in response to biotic stimuli and immune response were altered in MSCs exposed to IL-1β compared to control cells. In particular, genes essential for neutrophil recruitment were significantly upregulated after IL-1β exposure. The functional in vitro studies further validated these results, demonstrating that MSCs exposed to IL-1β had a significantly higher neutrophil recruitment capacity compared to unstimulated MSCs. Finally, inhibition of the NF-kB pathway resulted in a significant decrease of the MSC’s capacity to recruit neutrophils to levels similar as to the unstimulated control MSCs.

**Conclusion:**

These data provide mechanistic insight into how inflammatory factors present in the host microenvironment might affect the interaction between MSCs and immune cells. This further highlights the need to understand the MSC mode of action, and to map out how the MSC fate might change in different host environments after administration.

**Supplementary Information:**

The online version contains supplementary material available at 10.1186/s13287-026-05029-x.

## Background

Acute respiratory distress syndrome (ARDS) is an acute and complex syndrome characterized by accumulations of bilateral pulmonary infiltrates, noncardiogenic pulmonary edema, and acute hypoxemia [1, 2]. Despite current treatment strategies focusing on lung-protective ventilation, prone-positioning, and other supportive care, the mortality rate in ARDS patients remains high, and long-term morbidity among survivors is common [1, 2].

An uncontrolled immune response, with neutrophil accumulation in the lungs, is central to the pathogenesis of ARDS, and therefore novel treatment strategies with a capacity to attenuate this ramped immune response are needed [3, 4]. One such approach is the use of mesenchymal stromal cells (MSC) as cell-based therapy. Promising pre-clinical data using MSCs to treat acute lung injury in animal models has stimulated an increased interest and a rapidly growing number of clinical trials using MSCs for ARDS patients [5, 6]. The SARS-CoV-2 pandemic further accelerated the number of completed MSCs trials [7]. However, despite overall improvements observed with MSC treatments, particularly in COVID-19-associated ARDS, the question of why not all trials consistently demonstrate benefits remains [8, 9]. This variability is likely multifactorial. Studies, including our own, suggest that the therapeutic effect and viability of the MSCs are significantly affected by the host environment encountered after administration, including immune activation and inflammatory status [10–12]. However, differences in MSC origin, donor variability, number of cells, timing and administration route, manufacturing and cryopreservation procedures, may all contribute to the inconsistent therapeutic outcomes in clinical trials.

Interleukin-1β (IL-1β) is a pro-inflammatory cytokine [13] that has been linked to the development and progression of ARDS [null]. Interestingly, our previous work demonstrated that the presence of higher concentrations of IL-1β in bronchoalveolar lavage fluid samples (BALF) from ARDS patients and healthy control subjects could be used to predict activation of MSCs, leading to an increased secretion of a spectrum of pro-inflammatory cytokines [12]. However, the exact mechanisms through which IL-1β alters the MSC function and its interaction with the host immune cells remains unknown. Therefore, this study aimed to investigate how IL-1β stimulation alters the gene expression profile, protein secretion pattern, and neutrophil recruitment capacity of human bone marrow-derived MSC (BM-hMSC) in vitro, compared to unstimulated controls.

## Methods

### Cell isolation, expansion, and IL-1β stimulation

BM-hMSCs were isolated from one bone marrow aspirate obtained from a consenting healthy donor at the Hematology Department, Skåne University Hospital Lund, Sweden. Bone marrow mononuclear cells were isolated as described previously PMID: 31371413 by Hongzhe Li et al. BM-hMSCs were also purchased by PromoCell (cat# C-12974, lot# 472Z023.1 and lot# 439Z037.1) and Lonza (cat# PT-2501, batch# 0000669508). BM-hMSCs were routinely cultured in DMEM (cat# 61,965–026, Thermo Fisher) supplemented with 1% Antibiotic Antimycotic Solution (cat# A5955, MERCK) and 20% fetal bovine serum (cat# SH3008803, Cytiva) or 20% Fetal Clone 3 (FCIII, cat# SH3010903, Thermo Fisher) in standard tissue culture incubators. Cells at passages 5–6 were used in all experiments.

For exposures, BM-hMSCs were seeded into 6-well plates (100 000 cells/well, *i.e.* 10 417cells/cm^2^), 24-well plates (40 000 cells/well, *i.e.* 21 053 cells/cm^2^), or 96-well plates (10 000 cells/well, *i.e.* 31 250 cells/cm^2^) and left to adhere overnight in normal growth medium. After adherence, medium was removed, cells washed with Dulbecco’s phosphate-buffered saline (DPBS), and cells were cultured for 24 h in serum free DMEM. Following synchronization, medium was removed, and cells were stimulated with IL-1β (20 ng/ml in DPBS with 0.1% Bovine Serum Albumin, cat# 208-IL-010, R&D Systems) for 1 h (RNA-sequencing) or 24 h. Unstimulated control BM-hMSCs were exposed to serum free DMEM only. After 24 h stimulation, medium was removed, cells were washed two times with DPBS, and 500 μl serum free DMEM was added per well, and cells were incubated for 24 h before conditioned medium (CM) was collected. Viability was measured using commercial LDH kit (cat# 04744926001, Roche) according to manufacturer’s instructions and metabolic activity was measured using the commercial WST-1 kit (cat# 05015944001, Sigma Aldrich) according to manufacturer’s instructions. For the LDH assays, the lysis buffer provided in the kit served as the positive control. For the inhibition studies, BM-hMSCs were stimulated with 10 μM BAY 11–7082 (Cat # 196,871, Millipore) in combination with IL-1β for 24 h.

### RNA isolation and sequencing analysis

After 1 h IL-1β stimulation (n = 3), or serum free DMEM (n = 3), BM-hMSCs were washed two times with DPBS and cells were detached using TrypLE (cat# 12,604–013, Gibco). Cell suspension was centrifuged, and cell pellet was frozen at -80 °C. Total RNA was extracted using Qiagen RNeasy mini kit (Qiagen) and the RNA concentration was measured using a Qubit fluorometer. The RNA library was prepared using Truseq stranded mRNA LP kit (Illumina) according to manufacturer’s instructions using 350 ng of total RNA as input. One extra 1X bead clean of the final library was performed. Samples were sequenced using NovaseqX plus (Illumina) with loading concentration of 150 pM and read lengths 151-8-8-151. The raw RNA sequencing data were aligned to the human genome using STAR [14] and quantified using Salmon [15]. Transcript-level data were imported, and gene counts were scaled using trimport (v.4.17.0,(16)) on R (v. 4.5.1). DESeq2 (v.1.48.1, [17]) was used for gene count normalization and differential expression analysis. Pathway enrichment analysis was performed on significantly upregulated genes (log2foldchange > 2 and adjusted p-value < 0.05) using ClusterProfiler (v.1.36.0, [18]) based on the Biological Process terms from Gene Ontology [19–21]. Rstudio (v.2025.05.1 + 513) was used to generate plots alongside the packages ComplexHeatmap (v.2.24.1), ggplot2 (v.3.5.2) and EnhancedVolcano (v.1.26.0). The adjusted p-values were calculated using DeSeq2 utilizing the Wald test with Benjamini-Hochberg (FDR) correction.

### Cytokine analyses

Conditioned medium was centrifuged at 1000×g at RT for 10 min. For the initial analysis of cytokines, we performed a multiplex profiling of conditioned medium from BM-hMSCs (from a single donor) exposed to IL-1β and unstimulated cells using the Olink Target 96 Immuno-oncology panel (v. 3113, Supplementary Table 1). Based on the results from the initial Olink screening and bulk RNA sequencing data, selected proteins were further analyzed in CM from IL-1β stimulated and control BM-hMSCs isolated from two-four additional donors using ELLA instrument (Bio-techne) or ELISA. All samples were diluted 1:1 and the analytes were measured using custom made cartridges that included CCL2, CXCL5, and CXCL8 (Bio-techne) or measured by Quantikine ELISA (cat# D8000C, R&D Systems) with 1:1 dilution. CXCL1 was measured in diluted 1:1 or undiluted CM samples using Quantikine ELISA (cat# DGR00B, R&D Systems). Values that were higher than the standard curve were extrapolated, and the extrapolated values are presented as median. For measurements below standard curve, the values were set to 0.

### Neutrophil migration assay

The neutrophil migration assay was performed using a transwell system (Corning). Here, 75 000 human dermal microvascular endothelial cells (HMEC-1, cat# CRL-3243, ATCC) were seeded on top of the 5 μm pore size membrane and allowed to attach for 72 h in MCDB-131 medium supplemented with 10% FBS, 10 ng/ml hEGF, non-essential amino acids, sodium pyruvate, and PenStrep. After 72 h, the confluency and the membrane coverage of the HMEC-1 was assessed by light microscopy.

Neutrophils were isolated from peripheral blood collected from four healthy consenting donors as previously described [22]. Briefly, blood was collected in EDTA tubes, diluted in 0.9% NaCl, added to a Lymphoprep density gradient, and centrifuged according to manufacturer’s instructions. The red blood cell fraction was collected and mixed with 1.5% Dextran in 0.9% NaCl. Following centrifugation, the neutrophil containing supernatant was collected and resuspended in DMEM. The neutrophils cells were counted, and purity was checked using an XN-350 differential hematology analyzer (Sysmex Corporation).

After confirmation of HMEC-1 coverage on the transwell membrane, the insert was washed. Following PBS wash, the insert was moved to a fresh cell culture plate containing CM from the three BM-hMSC conditions: unstimulated controls, IL-1β stimulated cells, and IL-1β + NF-kB inhibitor stimulated cells. On top of the insert, 10 × 10^4^ neutrophils in 100 μl were added per well, and the cells were incubated for 2.5 h. After incubation, the inserts were discarded and the cells were collected from the bottom of the wells and stained with CD66b AF647 (clone G10F5, cat# 561,645, Biolegend). The number of migrated cells were counted using a cytoflex flow cytometer (Beckman Coulter).

### Western blot

For Western blot, BM-hMSCs were seeded into 6-well plates as previously described. Cell lysates were collected from the three BM-hMSC conditions: unstimulated controls, IL-1β stimulated cells, and IL-1β + NF-kB inhibitor stimulated cells. For the IL-1β + NF-kB inhibitor exposed cells, BM-hMSCs were treated with 10ul BAY 11–7082 inhibitor 30 min prior IL-1β stimulation (20 min). Following stimulation, cells were washed two times with ice cold DPBS, and cells were lysed with RIPA buffer containing 1X protease inhibitor cocktail (cOmplete, Roche) and 1X phosphatase inhibitor cocktail (PhosSTOP, Roche). Lysates were centrifuged for 10 min at 14,000 rcf at 4 °C. Samples were prepared in Laemmlis buffer and 10ug protein per sample were loaded and separated by electrophoresis on Mini-Protean TGX Stainfree 4–12% gels (Biorad, cat# 4,568,084) at 200 V for approximately 30 min. The protein was blotted on a Nitrocellulose membrane (Biorad, cat# 1,704,158) using the Trans Blot Turbo System. Following blocking with Every Blot Blocking buffer at RT for 20 min, membranes were stained with antibodies against Phospho-NF-kappaB p65 (Cell signaling, cat# 3033, diluted 1:1000) or NF-kappaB p65 (Cell signaling, cat# 8242, diluted 1:1000) in 5% BSA overnight. After the washing steps, membranes were stained with a secondary goat anti-rabbit antibody (cat# AB97051, diluted 1:10,000) in 2% BSA for 1 h. Following washing, membranes were incubated 5 min with Clarity Western ECL Substrate (Biorad, cat# 170–5061) and visualized using the ChemiDoc Touch Imaging System (Biorad). Phospho-p65 levels were quantified and normalized to total p65 using Image Lab software (Biorad, version 6.1). Data are expressed relative to control, which was set to 1.

### Statistical analyses

For normally distributed results, an unpaired t-test with Welch’s correction was used to assess differences between two groups. For non-normally distributed data, a Mann–Whitney test was used to assess differences between two groups. A one-way repeated measures (RM) ANOVA with Tukey’s multiple comparisons test was used to assess differences between three groups in the neutrophil migration assay. Statistical analyses were performed using GraphPad Prism software version 10.5.0. p-values ≤ 0.05 were considered significant.

## Results

### IL-1β significantly increase metabolic activity in BM-hMSCs

To initially determine if IL-1β were toxic to BM-hMSCs, we exposed cells to 20 ng/ml IL-1β for one or 24 h. Importantly, BM-hMSCs tolerated IL-1β as determined by lactate dehydrogenase (LDH) release. The levels of LDH released by the IL-1β exposed BM-hMSCs were significantly lower than the unstimulated control BM-hMSCs (p = 0.0006, Supplementary Fig. 1A and C). Moreover, a significant increase in metabolic activity was observed in IL-β exposed BM-hMSCs compared to unstimulated control cells (p < 0.0001, Supplementary Fig. 1B).

### IL-1β alters BM-hMSCs gene expression profile compared to unstimulated cells

**Fig. 1:**
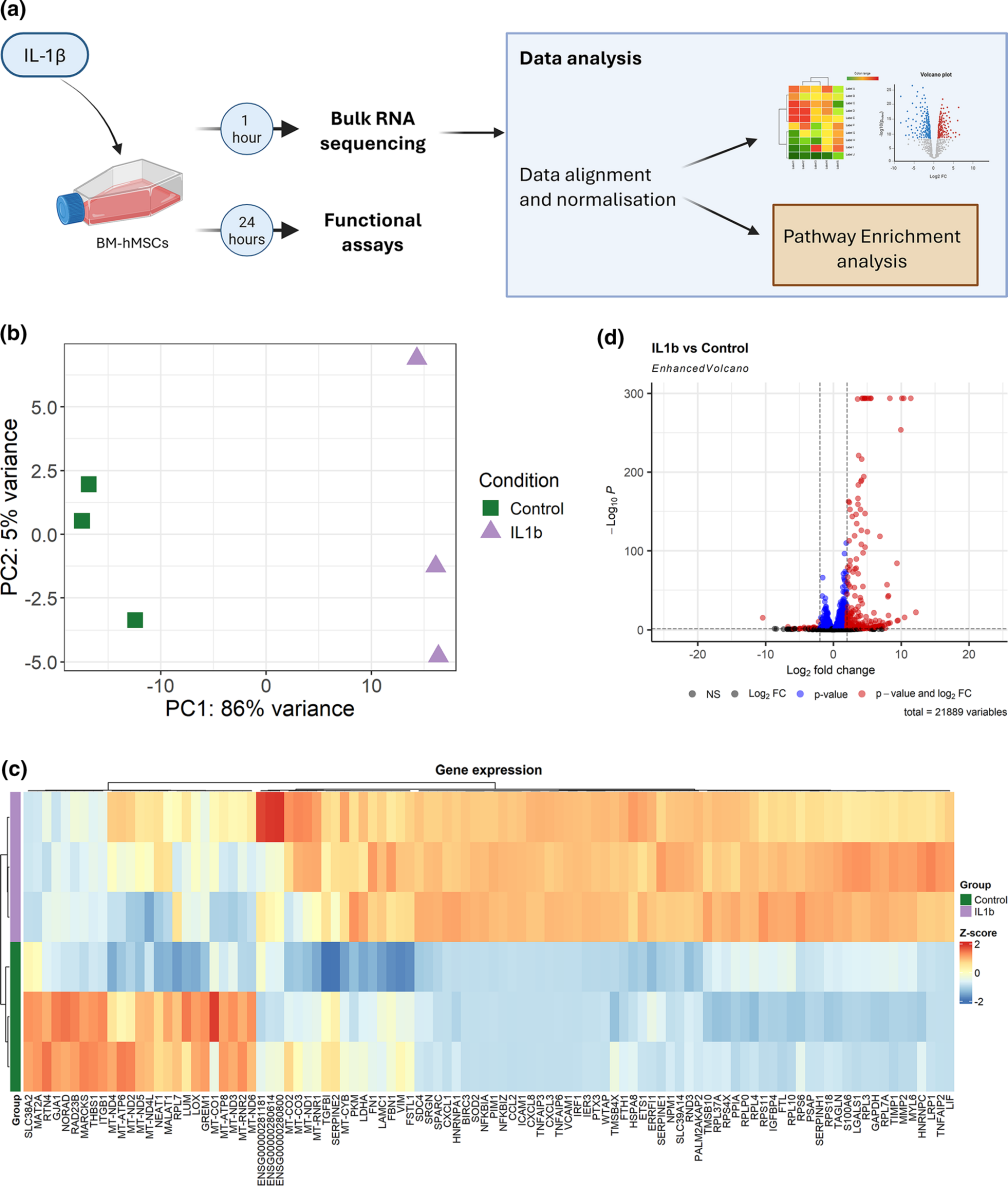
IL-1β stimulation changes the transcriptomic profile of BM-hMSCs. Schematic of experimental design **A**. BM-hMSCs were exposed to IL-1*β* (3 replicates, 1 donor) or left unstimulated (3 replicates, 1 donor) for one hour, followed by bulk RNA sequencing. PCA plot was used to show the variance between the unstimulated control (green) and IL-1*β* exposed (purple) samples **B**. Heatmap displaying the Z-score of the top 100 varying genes across all samples. Red color indicates higher expression of the genes and blue color indicates decreased expression **C**. Volcano plot of differentially expressed genes, showing their log2 fold change (X-axis) and -log10 adjusted p-values (Y-axis) **D**. IL-1*β* Interleukin-1β, BM-hMSCs, Bone marrow derived human mesenchymal cells, *PCA* Principal component analysis, FC Fold change, *NS* not significant. Figure 1A was created using Biorender.com

**Fig. 2:**
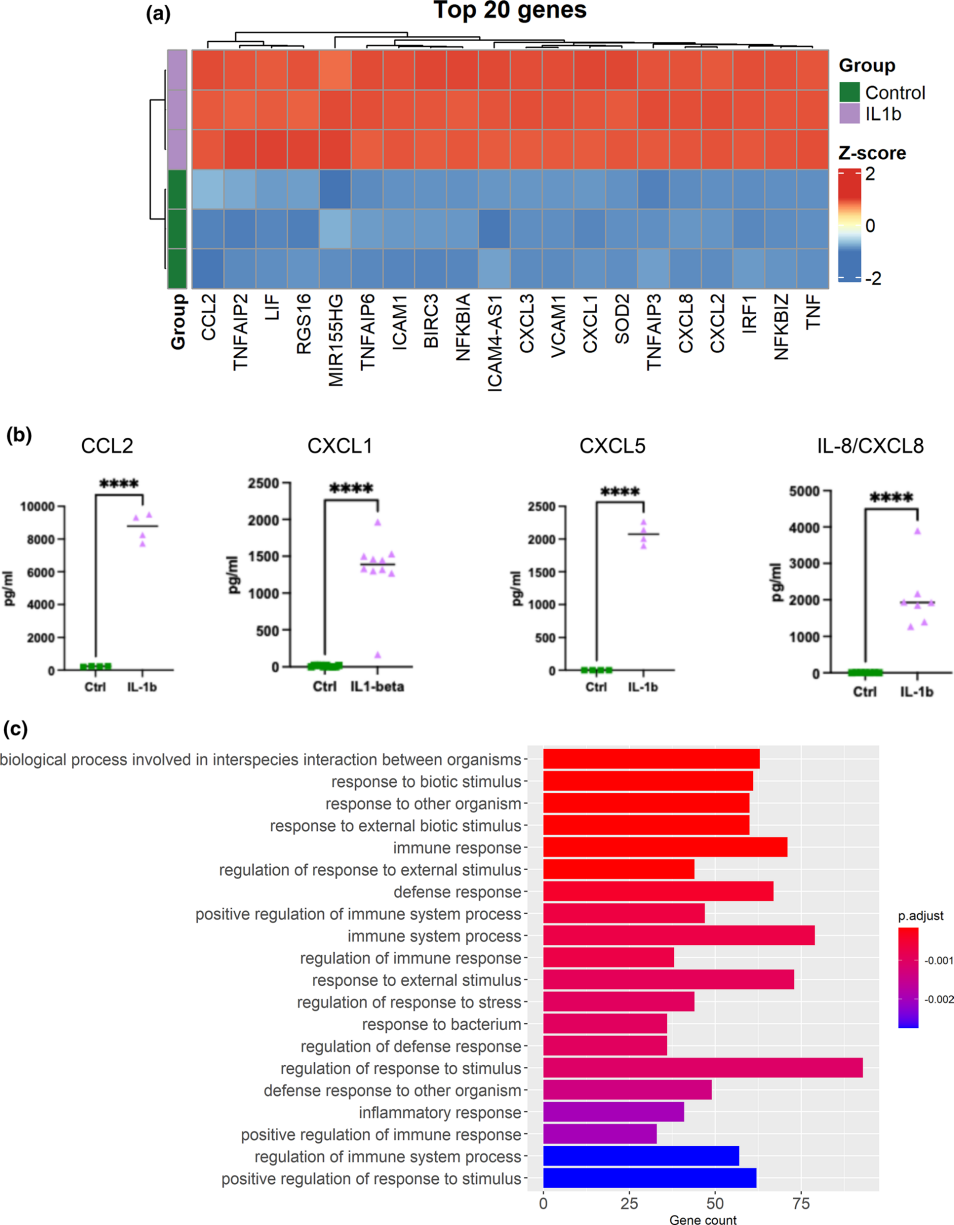
*IL-1β* stimulation increases expression of neutrophil recruitment and NF-kB signaling genes by BM-hMSCs. Heatmap clustering of the Z-score of the top 20 differentially expressed genes between unstimulated control BM-hMSCs (3 replicates, 1 donor, green) and IL-1*β* stimulated BM-hMSCs (3 replicates, 1 donor, purple). Red color indicates higher expression of the genes and blue color indicates decreased expression **A**. Protein expression of CXCL1 (10 replicates, 4 donors), CCL2 (4 replicates, 2 donors), CXCL5 (4 replicates, 2 donors), and CXCL8/IL-8 (7 replicates, 3 donors) in BM-hMSCs secretome measured by ELLA or ELISA. Data are presented as median, and statistical analysis was performed using unpaired t-test with Welch’s correction **B**. Bar plot of the top 20 significant Gene Ontology (GO) biological processes representing IL-*1β* induced upregulated differentially expressed genes **C** IL-1*β,* Interleukin-1β; BM-hMSCs, Bone marrow derived human mesenchymal cells; CCL2, chemokine (C–C motif) ligand 2; CXCL1, chemokine (C-X-C motif) ligand 1; CXCL2, chemokine (C-X-C motif) ligand 1; CXCL8, chemokine (C-X-C motif) ligand 8; ****, p < 0,0001

To ascertain the effects of IL-1β on BM-hMSC gene expression, bulk RNA sequencing was performed. An overall schematic of the study is presented in Fig. 1A. Principal component analysis (PCA) of normalized gene count variance revealed clustering of samples by stimulation (Fig. 1B). A heat map demonstrates the gene counts Z-scores of the stimulated and unstimulated BM-hMSCs (Fig. 1C). These data clearly demonstrate that IL-1β activates BM-hMSCs changing their gene expression profile already at 1 h of exposure. 351 genes (log2 Fold Change > 2 and adjusted p < 0.05) were found to be upregulated in the IL-1β exposed BM-hMSCs compared to the unstimulated control cells, and 36 genes (log2 Fold Change < -2 and adjusted p < 0.05) were found to be downregulated. As shown in Fig. 1D, there were a group of genes that clustered in the top right corner of the volcano plot, with very high log2 fold change, and low log10 p-values. To explore these genes more in detail, we plotted the top 20 differentially expressed genes using a heat map (Fig. 2A). Interestingly, among the top genes we found chemokine (C–C motif) ligand 2 (CCL2), chemokine (C-X-C motif) ligand 1 (CXCL1), chemokine (C-X-C motif) ligand 5 (CXCL5), and chemokine (C-X-C motif) ligand 8 (CXCL8) which are all genes involved in neutrophil recruitment. Other genes in the top 20 list, were genes involved in regulation of the nuclear factor-kappa-B (NF-kB) signaling pathway, such as NF-kB Inhibitor Alpha (NFKBIA) and NF-kB Inhibitor Zeta (NFKBIZ) (Supplementary Fig. 2). To further validate these observations, the protein level of CCL2, CXCL1, CXCL5, and CXCL8/IL8 in the secretome from BM-hMSCs were determined using ELLA and ELISA. These results demonstrated a significant upregulation of CCL2 (p < 0,0001), CXCL1 (p < 0,0001), CXCL5 (p < 0,0001), and CXCL8 (p < 0,0001) in IL-1β exposed BM-hMSCs compared to unstimulated controls (Fig. 2B).

### Exposure to IL-1β results in upregulation of genes involved in response to biotic stimuli and immune response

**Fig. 3:**
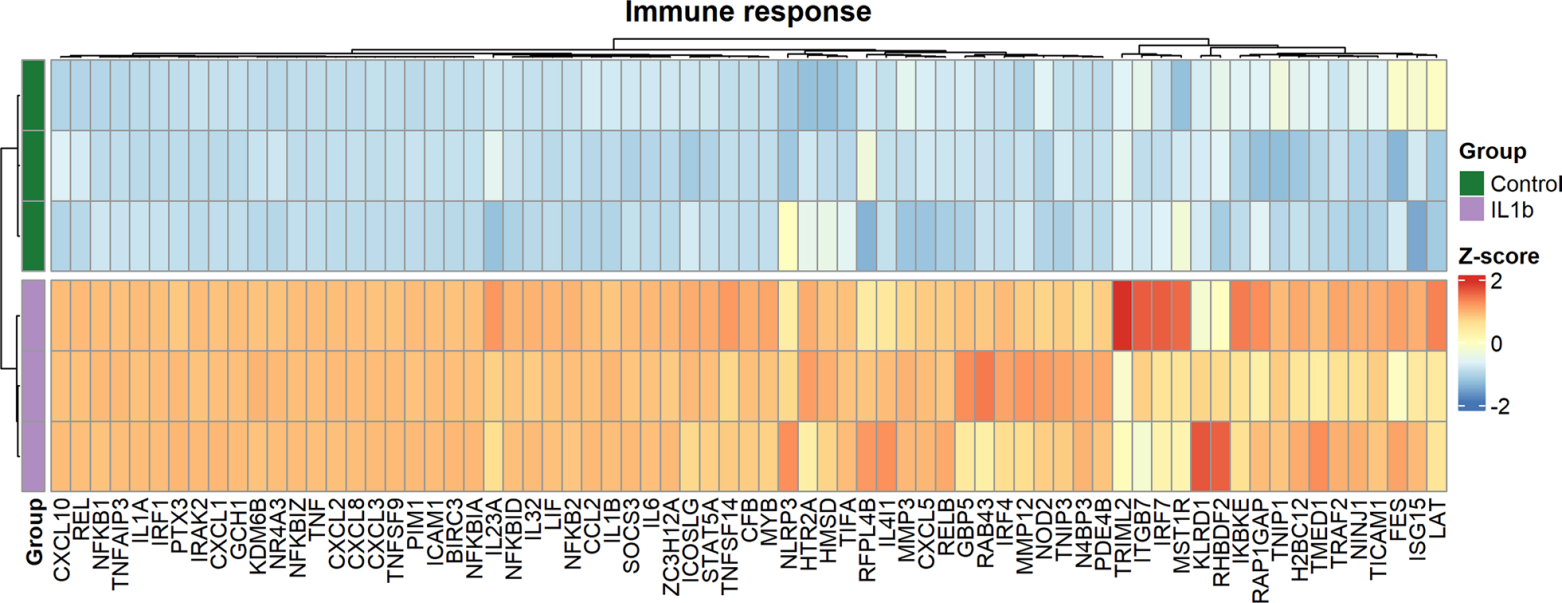
Neutrophil recruitment and NF-kB signaling genes involved in the immune response of BM-hMSCs*.* Heatmap displaying the Z-score of differentially expressed genes involved in the immune response gene ontology biological process between unstimulated control BM-hMSCs (3 replicates, 1 donor, green) and IL-1*β* stimulated BM-hMSCs (3 replicates, 1 donor, purple). Red color indicates higher expression of the genes and blue color indicates decreased expression. IL-1*β* Interleukin-1β, BM-hMSCs Bone marrow derived human mesenchymal cells

To better understand and describe the biological function of the differentially expressed genes, we performed a Gene Ontology (GO) enrichment analysis of the over-represented gene list. Here, we found that IL-1β altered genes belonged to the biological process involved in interspecies interaction between organisms, response to biotic stimulus, response to other organisms, response to external biotic stimulus, and immune response compared to unstimulated BM-hMSCs (Fig. 2C). To further explore which pathways to focus on, we prepared a cluster net plot of the top 5 GO terms and net of the associated genes (Supplementary Fig. 3). The cluster net plot demonstrated clearly that the top 4 pathways clustered together and that the immune response node clustered separately. Since, the immune response node clearly had a different gene network compared to the other 4 pathways in combination with the biological relevance in ARDS pathology, we decided to focus our study on the genes included in the immune response node. To further explore the immune response pathway, we plotted the genes involved in the pathway on a heatmap (Fig. 3). The genes CCL2, CXCL1, CXCL5, and CXCL8, that were identified as a cluster in the volcano plot (Fig. 1D), were found to be involved in the immune response pathway. These are all genes involved and crucial for neutrophil recruitment, which is relevant considering the neutrophils involvement in the ARDS pathogenesis [1] and our previous findings [12]. Interestingly, genes involved in regulating signaling pathways especially NF-kB signaling pathway such as REL, NFKB1, NFKB2, RELB, NFKBIA, NFKBIZ, TNFAIP3, TRAF2, and TIFA (Fig. 3) were also differentially upregulated in the IL-1β exposed BM-hMSCs.

### Exposure to IL-1β results in increased neutrophil recruitment partly via the NF-kB signaling pathway

**Fig. 4:**
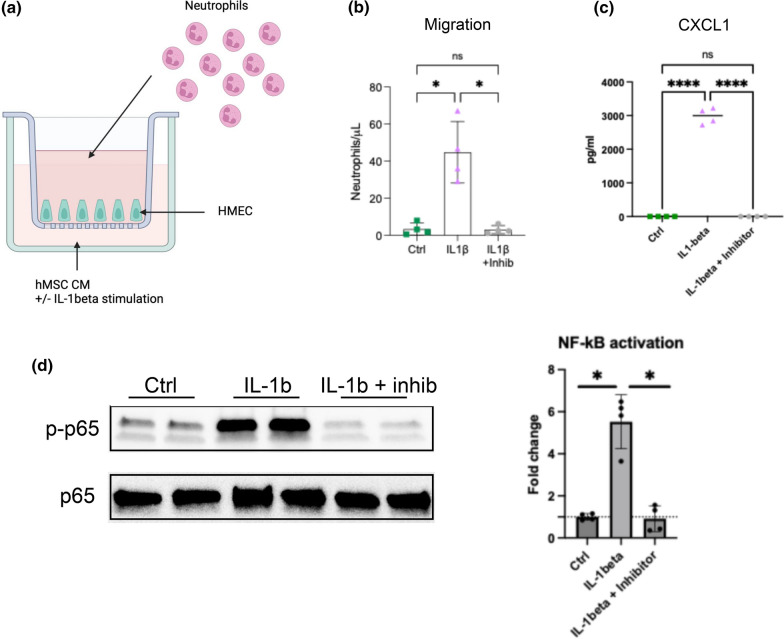
IL-1β stimulated BM-hMSCs increased neutrophil recruitment partly via the NF-kB signaling pathway. Schematic of experimental design of the neutrophil migration assay. Illustration was created with BioRender.com **A**. Number of neutrophils that migrated through the transwell membrane from the top well to the bottom towards control conditioned medium, conditioned medium from IL-1β stimulated BM-hMSCs, and conditioned medium from IL-1β with the NF-kB inhibitor stimulated BM-hMSCs. The experiment was performed using neutrophils from four different donors (each donor represent one data point) and the experiments were performed on three different days. **B**. Protein expression of CXCL1 was measured in conditioned medium from unstimulated BM-hMSCs (4 replicates, 1 donor, green), IL-1β stimulated BM-hMSCs (4 replicates, 1 donor, purple), and ILβ stimulated BM-hMSCs with 10 μM BAY 11–7082 (4 replicates, 1 donor, grey) **C**. Phospho-p65 levels were quantified and normalized to total p65. Data are expressed relative to control, which was set to 1 **D**. IL-1β Interleukin-1β BM-hMSCs Bone marrow-derived human mesenchymal cells, Ctrl control, CXCL1 chemokine (C-X-C motif) ligand 1, ns not significant; * p < 0,05; ****, p < 0,0001

Based on the findings in the immune response pathway, we next wanted to assess the IL-1β stimulated BM-hMSCs role on neutrophil recruitment. Here we utilized a transwell system to assess if conditioned medium from IL-1β exposed BM-hMSCs increased the neutrophil recruitment capacity compared to the unstimulated BM-hMSCs (Fig. 4A). These results, suggest that IL-1β stimulated BM-hMSCs significantly increased the neutrophil migration compared to unstimulated controls (p = 0.0272, Fig. 4B). Next, we wanted to assess if this neutrophil recruitment capacity was driven via the NF-kB pathway since several genes coding for proteins involved in the NF-kB signaling pathway were identified in the RNA sequencing data. Therefore, the neutrophil migration experiment was performed with and without adding the NF-kB inhibitor BAY 11–7082 to the system. To first validate that the inhibitor worked, we measure the levels of CXCL1 in conditioned medium from BM-hMSCs exposed to IL-1β with or without the NF-kB inhibitor. These results demonstrated that, BM-hMSCs exposed to IL-1β had a significantly increased expression of CXCL1 in the medium compared to the unstimulated cells (p < 0.0001, Fig. 4C). In contrast, in conditioned medium from BM-hMSCs exposed to IL-1β in combination with the NF-kB inhibitor, a significant decrease was observed compared to BM-hMSCs exposed to IL-1β only (p < 0.0001). No significant difference between the unstimulated cells and the BM-hMSCs exposed to both IL-1β and the inhibitor (p > 9999) was observed (Fig. 4C). To further validate NF-kB inhibition, Western blot analysis was performed, confirming reduced NF-kB pathway activation in BM-hMSCs treated with IL-1β in the presence of the BAY 11–7082 inhibitor (Fig. 4D). Together, these results ensured us that the inhibitor worked well, and we therefore performed the neutrophil migration assay with and without the NF-kB inhibitor. NF-kB inhibition significantly inhibited the neutrophil recruitment capacity compared to IL-1β exposed BM-hMSCs only (p = 0.0282). Importantly, no significant difference was found between unstimulated BM-hMSCs and the cells exposed to both IL-1β and the NF-kB inhibitor (p = 0.9118). Taken together, these data suggest that IL-1β stimulation results in an increased neutrophil recruitment via the NF-kB signaling pathway.

## Discussion

We have previously found that high levels of IL-1β in BALF samples from ARDS patients and healthy controls correlated with activation of BM-hMSCs. Exactly through which mechanisms this is done and what effect it has on BM-hMSCs interaction with the host immune cells remain unknown. In this study we found that BM-hMSCs exposed to IL-1β had an increased expression of genes involved in immune responses. Moreover, we were able to demonstrate that IL-1β increased the capacity of BM-hMSCs to recruit neutrophils, a response that was inhibited by addition of a NF-kB inhibitor, suggesting that this neutrophil recruitment potential was, at least partly, via the NF-kB signaling pathway.

IL-1β is a pro-inflammatory cytokine included in a larger protein complex called inflammasome, and has previously been linked to a more severe form of ARDS with a worse progression and increased mortality [23]. Several studies, including our own, have demonstrated that IL-1β can activate MSCs, resulting in altered immunosuppressive properties [12, 24]. Understanding exactly how MSCs are changed when exposed to IL-1β is important, especially in ARDS where there are several different subgroups with different microenvironments [25]. A study by Fan et al. demonstrated that umbilical cord-derived MSCs stimulated with 10 ng/ml IL-1β for 48 h were able to reduce dextran sulfate sodium (DDS)-induced colitis in mice better than control MSCs [24]. Interestingly, the study also suggested that the IL-1β exposed MSCs changed the balance of the immune cells, focusing mainly on macrophage subtypes and Foxp3^+^ Treg cells, in the DDS-induced colitis mouse model suggesting that the presence of IL-1β in the host might be beneficial at least in colitis [24]. Our data suggest that BM-hMSCs exposed to IL-1β had a significantly increased capacity to recruit neutrophils. In contrast to the findings in the Fan study, this is most likely not a beneficial outcome in a neutrophil driven inflammatory disease, since this would most likely further promote tissue damage and lung dysfunction.

The RNA sequencing data clearly demonstrated that IL-1β induced expression of genes involved in the NF-kB pathway, suggesting that NF-kB is one of the main regulators of the inflammatory response [26]. This was further supported by the functional neutrophil migration assay, in which we observed that the recruitment of neutrophils was completely suppressed when IL-1β -exposed BM-hMSCs were stimulated with the NF-kB inhibitor. This suggests that when MSCs are exposed to IL-1β they are switching to a more proinflammatory phenotype, an observation that is supported by the increased secretion of CCL2, CXCL1, CXCL5, and CXCL8/IL8 seen from MSCs exposed to IL-1β. This suggests that MSCs administered into a host environment with high levels of IL-1β, such as in some of the ARDS patients, might create a worse situation leading to even more neutrophils being recruited to the inflamed lungs. This could be one of possible explanations for the heterogenous clinical outcomes that have been observed in ARDS clinical trials [8, 27–29]. However, the significance of this finding in vivo needs to be further studied. In addition, donor-to-donor variability of BM-hMSCs should be considered, as it is likely to influence the secretion of inflammatory mediators such as CCL2, CXCL1, CXCL5, and CXCL8/IL-8 [30]. In our study, variability in protein secretion levels between different BM-hMSC donors were observed, however, all donors consistently exhibited a strong response to IL-1β exposure. This suggests that, despite the donor-to-donor variation, the phenotypic shift towards a pro-inflammatory phenotype induced by IL-1β is a reproducible feature of BM-hMSCs.

This study has several strengths. First, BM-hMSCs used in this study were both isolated in house from bone marrow aspirates obtained from healthy donors, allowing us to have full control over isolation method and culture conditions, and from a commercially available company. Another strength is that we used primary human neutrophils for the migration assay. Therefore, the findings in this study likely mirrors clinical settings. However, one caveat is that the NF-kB inhibitor used in this study to inhibit the neutrophil recruitment capacity is a broad-spectrum inhibitor with multiple-targets [31]. However, the functional data in combination with the RNA-sequencing data suggests to us that it is likely, at least partly, that the neutrophil recruitment occurs via NF-kB signaling. Although further studies are needed to rule out if this could also be via other signaling pathways. Another limitation of this study design is that only one dose of 20 ng/ml IL-1β was used. This concentration was selected based on the literature, where concentrations in the range of 20-25 ng/ml are commonly used [32–34]. In preliminary experiments, we used lower concentrations of IL-1β (5–20 ng/ml), and we did not observe any significant differences between the lower and higher concentrations (Supplementary Table 1). One additional caveat of this study is the lack of in vivo experiments to validate the relevance of the results in ARDS. As such we can only speculate that IL-1β in ARDS patients could enhance the neutrophil recruitment capacity of administered MSCs and thereby worsen the ARDS disease progression and further in vivo studies are therefore needed.

## Conclusion

BM-hMSCs exposed to IL-1β show altered expression of genes and proteins associated with immune response and NF-kB signaling, and an increase in secreted cytokines that may contribute to neutrophil recruitment. These data suggest that IL-1β can modulate the inflammatory profile of MSCs. Our results thus highlight the importance to understand how host-derived mediators might influence the functional properties of administered BM-hMSCs.

## Supplementary Information

Supplementary material 1.

Supplementary material 2.

## Data Availability

The Olink data and the bulk RNA-seq datasets are included in the supplementary files.

## Abbreviations

ARDS: Acute respiratory distress syndrome
BALF: Bronchoalveolar lavage fluid samples
BM-hMSCs: Human bone marrow-derived MSCs
CCL2: Chemokine (C–C motif) ligand 2
CXCL1: Chemokine (C-X-C motif) ligand 1
CXCL5: Chemokine (C-X-C motif) ligand 5
CXCL8: Chemokine (C-X-C motif) ligand 8
GO: Gene Ontology
IL-1β: Interleukin-1β
LDH: Lactate dehydrogenase
MSC: mesenchymal stromal cells
NF-Kb: nuclear factor-kappa-B
NFKBIA: NF-kB inhibitor alpha
NFKBIZ: NF-kB inhibitor zeta
PCA: Principal component analysis
